# A change in temperature modulates defence to yellow (stripe) rust in wheat line UC1041 independently of resistance gene *Yr36*

**DOI:** 10.1186/1471-2229-14-10

**Published:** 2014-01-08

**Authors:** Ruth R M Bryant, Graham R D McGrann, Alice R Mitchell, Henk-jan Schoonbeek, Lesley A Boyd, Cristobal Uauy, Steve Dorling, Christopher J Ridout

**Affiliations:** 1 Department of Crop Genetics, John Innes Centre, Norwich Research Park, Norwich NR4 7UH, UK; 2 University of Sheffield, Western Bank, Sheffield S10 2TN, UK; 3 National Institute of Agricultural Botany, Huntingdon Road, Cambridge CB3 0LE, UK; 4 School of Environmental Sciences, UEA, Norwich NR4 7TJ, UK; 5 Department of Life and Medical Sciences, University of Hertfordshire, Hatfield, Hertfordshire AL10 9AB, UK; 6 Present address: Crop Protection Team, Crop and Soil Systems Group, SRUC, West Mains Road, Edinburgh EH9 3JG, UK

**Keywords:** Yellow rust, Plant defence, Temperature, *Yr36*, *Puccinia striiformis* f. sp *tritici*, HTAP

## Abstract

**Background:**

Rust diseases are of major importance in wheat production worldwide. With the constant evolution of new rust strains and their adaptation to higher temperatures, consistent and durable disease resistance is a key challenge. Environmental conditions affect resistance gene performance, but the basis for this is poorly understood.

**Results:**

Here we show that a change in day temperature affects wheat resistance to *Puccinia striiformis* f. sp *tritici* (*Pst*), the causal agent of yellow (or stripe) rust. Using adult plants of near-isogenic lines UC1041 +/- *Yr36*, there was no significant difference between *Pst* percentage uredia coverage in plants grown at day temperatures of 18°C or 25°C in adult UC1041 + *Yr36* plants. However, when plants were transferred to the lower day temperature at the time of *Pst* inoculation, infection increased up to two fold. Interestingly, this response was independent of *Yr36*, which has previously been reported as a temperature-responsive resistance gene as *Pst* development in adult UC1041 *-Yr36* plants was similarly affected by the plants experiencing a temperature reduction. In addition, UC1041 *-Yr36* plants grown at the lower temperature then transferred to the higher temperature were effectively resistant and a temperature change in either direction was shown to affect *Pst* development up to 8 days prior to inoculation. Results for seedlings were similar, but more variable compared to adult plants. Enhanced resistance to *Pst* was observed in seedlings of UC1041 and the cultivar Shamrock when transferred to the higher temperature. Resistance was not affected in seedlings of cultivar Solstice by a temperature change in either direction.

**Conclusions:**

*Yr36* is effective at 18°C, refining the lower range of temperature at which resistance against *Pst* is conferred compared to previous studies. Results reveal previously uncharacterised defence temperature sensitivity in the UC1041 background which is caused by a change in temperature and independently of *Yr36*. This novel phenotype is present in some cultivars but absent in others, suggesting that *Pst* defence may be more stable in some cultivars than others when plants are exposed to varying temperatures.

## Background

Rusts are among the most economically important and widespread diseases of wheat worldwide. Yellow (or stripe) rust, caused by *Puccinia striiformis* f. sp. *tritici* (*Pst*), is predominantly found in temperate regions and propagates mainly through asexual urediniospores produced in uredia on the leaf surface. *Pst* is becoming more prevalent, possibly due to the evolution of more aggressive isolates which have evolved to tolerate higher temperatures [1,2]. In parallel, several major sources of wheat resistance to *Pst* have broken down in recent years. These developments hasten the need to identify more effective and durable sources of resistance.

*Pst* urediniospores germinate on the wheat leaf surface, forming a germ tube that enters the plant through stomata. Once inside, a sub-stomatal vesicle (SSV) is formed within the stomatal cavity from which infection hyphae form. A haustorial mother cell is formed at the end of each infection hypha upon contact with a plant mesophyll cell. An infection peg breaches the plant cell wall forming a fungal feeding structure, known as a haustorium, within the cell. Further hyphae develop from the infection hyphae and proliferate throughout the leaf [3]. Approximately two weeks after the pathogen has entered the plant, visible symptoms can be seen in susceptible reactions as raised pustules, known as uredia forming on the leaf surface.

The effect of temperature on the performance of resistance genes is well-documented. Several quantitative disease resistance (QDR) genes, conferring enhanced resistance to *Pst* at either high or low temperatures, have been identified in wheat [4]. QDR is often described as adult plant or partial resistance, or as ‘slow rusting’. The effect of temperature on yellow rust resistance genes is well-known (reviewed in [5]). Seedlings of wheat cultivars with high temperature adult plant (HTAP) resistance are susceptible to all races of *Pst* at high and low temperatures. Adult plants of HTAP resistance cultivars are susceptible at low temperatures, but resistant at high temperatures. Yellow rust resistance genes *Yr36* and *Yr39* were originally designated as HTAP genes because exposure to higher temperatures was essential for their function at later plant growth stages [6,7]. However, *Yr36* was later shown to confer temperature-dependent resistance at all growth stages when exposed to temperatures over 25°C [8]. In contrast to HTAP genes, leaf rust resistance conferred by *Lr34/Yr18* is enhanced at lower temperatures [9-12]. *Lr34*/*Yr18* is a well-established resistance, being present in many wheat cultivars and occupying more than 26 million hectares in developing countries [13], whereas *Yr36* has more recently been introduced into many varieties worldwide through the introgression of the closely linked *Gpc-B1* gene [14-16]. In addition, several wheat cultivars have been identified as containing un-characterised yellow rust temperature–responsive resistance genes, suggesting that such genes are widely deployed in agriculture [17-19].

Plants have several layers of defence that provide protection against invading microbes. Plants recognise conserved microbial elicitors, known as pathogen (or microbe)-associated molecular patterns (PAMPs/MAMPs), through pattern recognition receptors (PRRs), initiating PAMP-triggered immunity (PTI). PTI activates inducible defences, including cell wall reinforcement, production of antimicrobial compounds and stomatal closure which are sufficient to repel or deter most invading microbes [20,21]. Virulent pathogens have evolved to suppress PTI, producing effector proteins that interfere with PRR function or downstream signalling components. Another layer of defence is provided by resistance (R) proteins that detect these effector proteins initiating effector-triggered immunity (ETI), a type of resistance typified by the hypersensitive response (HR) associated with host cell death [22]. Most R genes are predicted to encode proteins with nucleotide-binding site (NBS) and leucine-rich repeat (LRR) domains involved in effector recognition. However, cloning of the QDR genes *Yr36* and *Lr34* has revealed that these genes do not belong to the major NBR-LRR class of genes, suggesting that the mechanisms of resistances conferred by these genes could be fundamentally different from those of R genes [8,13].

At present, the basis for temperature-responsive resistance is poorly understood [10,12,13]. In this study, we investigate temperature-sensitivity in the wheat-yellow rust pathosystem using near isogenic lines (NILs) of the hexaploid wheat breeding line UC1041 *+/- Yr36*. Plants were grown under two temperature regimes which were maintained at 12°C during the dark period and 18°C or 25°C during the light period (hereby referred to as 12°C/18°C or 12°C/25°C). After inoculation, plants were either kept at the original temperatures they were grown under or transferred to the other temperature regime. Our findings reveal previously uncharacterised background sensitivity to temperature changes in UC1041 that acts independently of *Yr36*. Defence against *Pst* in adult UC1041-*Yr36* was affected by a temperature change in both directions and this effect was consistent in plants transferred up to 8 days prior to inoculation. Enhanced resistance when plants experienced an increase in temperature was observed in seedlings, and manifested as reduced hyphal colonisation compared to seedlings maintained at cooler day temperatures. We also show that this temperature sensitivity is genotype-dependent in wheat seedlings. Our work highlights the importance of temperature changes, rather than defined thresholds, when characterising disease resistance mechanisms in wheat. The results have potential to inform breeding strategies for the creation of wheat cultivars with more environmentally stable *Pst* resistance.

## Results

### A decrease in day temperature diminishes resistance to *Pst* independently of *Yr36*

**Figure 1:**
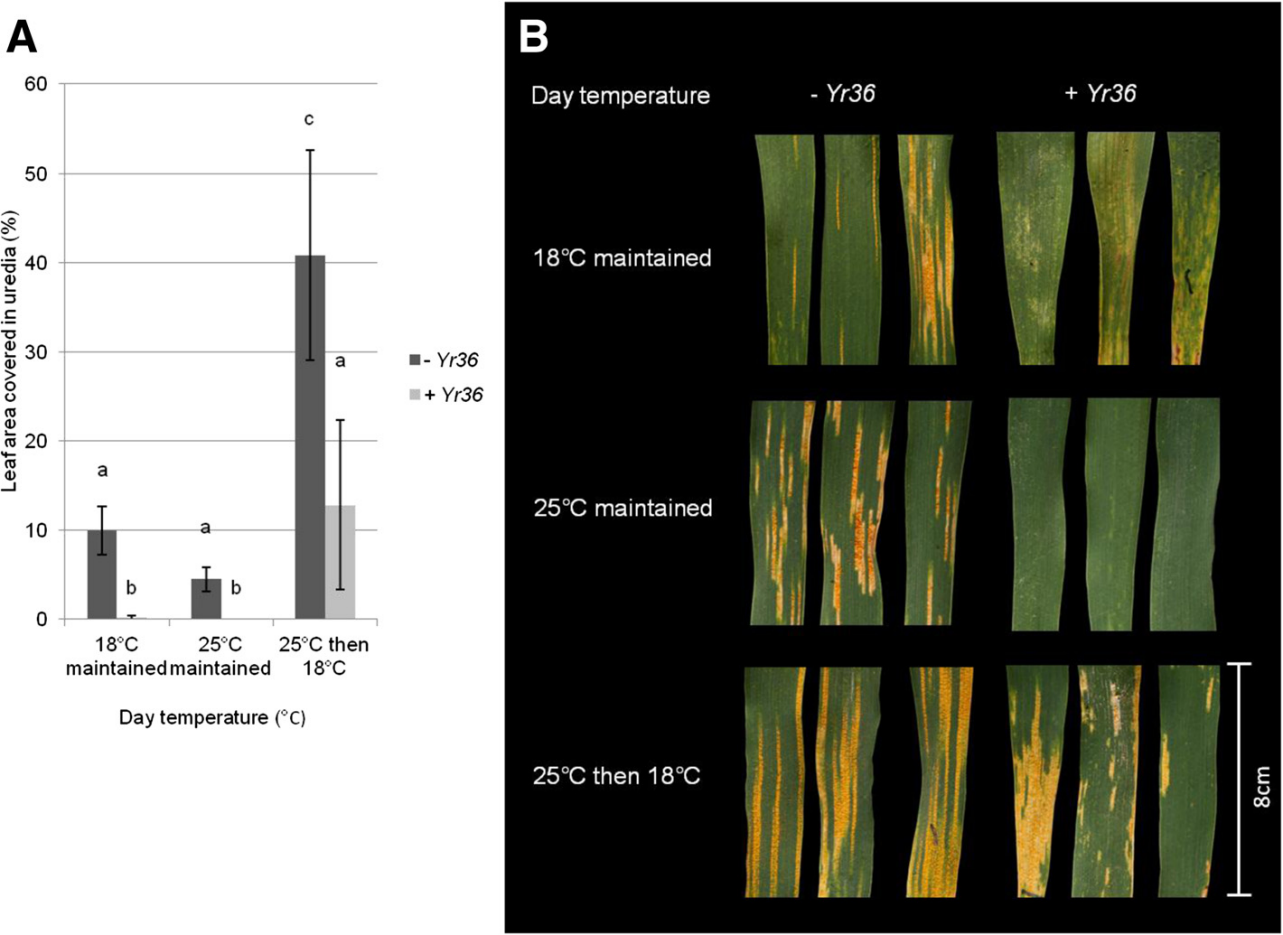
**The effect of changes in temperature on yellow rust resistance in adult plants of wheat lines UC1041 +/- *Yr36. *A)** Mean percentage (%) leaf area covered in pustules at 18 dpi on UC1041 - *Yr36* (dark grey) and UC1041 + *Yr36* (light grey) adult plants inoculated with *Pst* isolate 08/21 under three different temperature treatments. Mean values (± 1 standard error) were obtained from six biological replicates. Data was analysed using the general linear model and statistically significant differences are indicated by a different letter (*P <0.01*). **B)***Pst* symptoms at 18 dpi on flag leaf sections from plants at the three temperature treatments. Uredia can be observed within necrotic/chlorotic areas on the leaf surface.

*Yr36* conferred near-complete resistance in adult plants of UC1041 *+ Yr36* when plants were maintained in either the 12°C/18°C or the 12°C/25°C temperature regimes pre and post inoculation. However, *Yr36*-mediated resistance was significantly reduced in plants originally grown at 12°C/25°C and then transferred, post-inoculation to 12°C/18°C, uredia coverage being significantly higher (*P < 0.01*, Figure 1A,B). As expected, UC1041 *-Yr36* plants were less resistant than UC1041 *+ Yr36*, and there were no significant differences in disease levels between plants which were maintained at 12°C/18°C or 12°C/25°C pre and post-inoculation (Figure 1A,B). However, resistance in UC1041 *–Yr36* was also significantly reduced when adult plants were transferred from the 12°C/25°C to 12°C/18°C regime following inoculation, with leaf uredia coverage increasing up to two fold (*P < 0.01*). Changing temperatures therefore affects resistance in both UC1041 NILs independent of the presence of *Yr36*.

### A change in temperature affects *Pst* resistance up to 8 days pre-inoculation in UC1041

**Figure 2:**
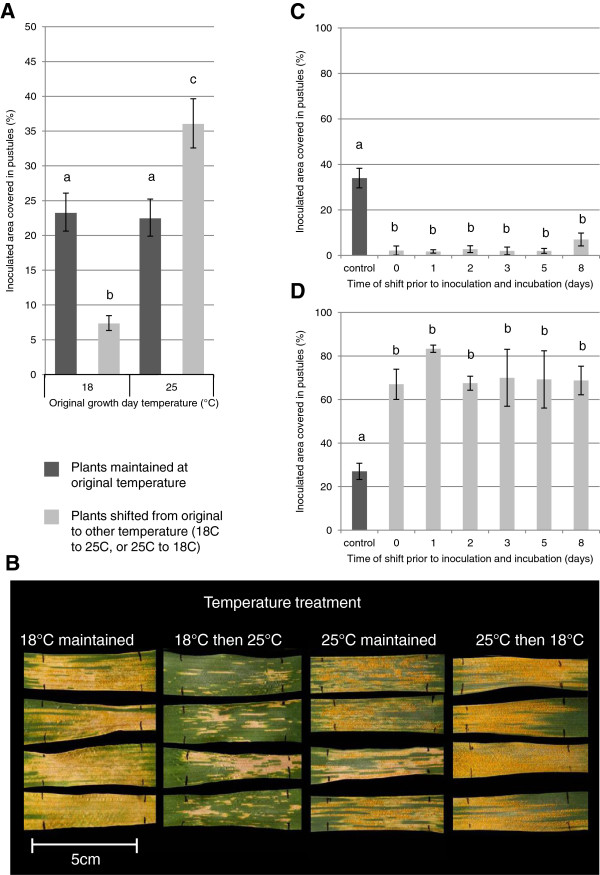
**The effect of changes in temperature, up to eight days before inoculation with *Pst, *on yellow rust resistance in adult plants of the wheat line UC1041 - *Yr36. *A)** Mean percentage (%) leaf area covered in uredia 18 dpi on adult plants inoculated with *Pst* isolate 08/21 kept at 12°C/18°C or 12°C/25°C pre and post inoculation (dark grey) or transferred to the other temperature regime post-inoculation (light grey). Mean values (± 1 standard error) were obtained from three independent experiments. Different letters indicate statistical significant differences (*P < 0.01*). **B)***Pst* symptoms on flag leaves at 18 dpi in the four temperature regimes. Uredia can be observed within necrotic/chlorotic areas. **C-D)** Mean percentage (%) leaf area covered in uredia at 18 dpi on adult plants inoculated with *Pst* isolate 08/21 and kept at the original temperature regime of **C)** 12°C/18°C or **D)** 12°C/25°C pre and post inoculation (dark grey) or shifted at various time points before inoculation to the other temperature regime (light grey). Mean values (± 1 standard error) were obtained from four biological replicates. Different letters indicate statistically significant differences (*P < 0.01*).

Initial observations with the UC1041 +/-*Yr36* NILs led us to further characterise the temperature-responsive *Pst* resistance of the UC1041 background. As previously observed, there was no significant difference in percentage uredia coverage of plants maintained pre and post inoculation at 12°C/18°C or 12°C/25°C (Figure 2A,B), whilst plants transferred from the higher to the lower temperature regime were less resistant (*P < 0.01*). Conversely, plants grown at 12°C/18°C and then transferred to 12°C/25°C post inoculation were more resistant, with significantly lower uredia levels (*P* < 0.001, Figure 2A,B). The extent of *Pst* colonisation varied between each of the three experiments, although similar amounts of spores were applied. Nevertheless, the trend in adult UC1041 -*Yr36* plants remained consistent in each experiment, with temperature decreases diminishing resistance and temperature increases resulting in enhanced resistance. UC1041 resistance can respond differently at 12°C/18°C and 12°C/25°C. At 12°C/25°C the uredia are surrounded by necrotic tissue (dead cells), indicative of a cell death/hypersensitive response (HR). The uredia are often surrounded by chlorotic tissue, the HR being weak. The shift from 12°C/18°C to 12°C/25°C may trigger a cell death response and result in a retardation of uredia formation.

Similar experiments were performed with adult plants transferred from one temperature regime to the other up to 8 days before inoculation. There were no significant differences in the reduced uredia levels between plants transferred from 12°C/18°C to 12°C/25°C at the time of inoculation compared to those transferred 1, 2, 3, 5 or 8 days prior to inoculation (Figure 2C). Similarly, adult plants transferred from 12°C/25°C to 12°C/18°C had comparable increased numbers of uredia regardless of whether they were transferred at the time of inoculation or 1, 2, 3, 5 or 8 days before inoculation (Figure 2D).

### A decrease in day temperature affects later stages of *Pst* colonisation in UC1041 seedlings

**Figure 3:**
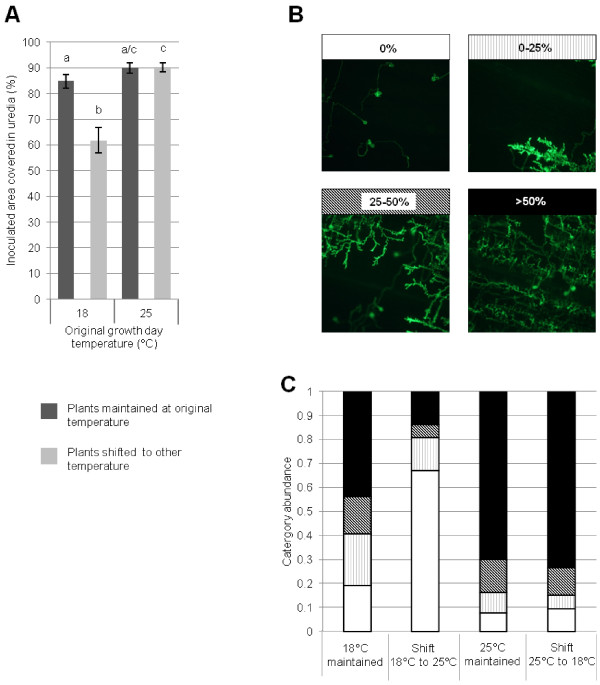
**The effect of changes in temperature on *Pst *development in seedlings of the wheat line UC1041 - *Yr36. *A)** Mean leaf area (%) covered in uredia 18 dpi on UC1041 -*Yr36* seedlings. Leaves were inoculated with *Pst* isolate 08/21 and kept at the original temperature regime of 12°C/18°C or 12°C/25°C pre and post inoculation (dark grey) or transferred to the other temperature regime post-inoculation (light grey). Mean values (± 1 standard error) were obtained from up to 50 fields of view in three independent experiments. Different letters indicate statistically significant differences (*P < 0.01*). **B-C)** Microscopic characterisation of *Pst* development as categorised by the extent of hyphal colonisation. **B)** Illustration of *Pst* hyphal abundance, with each category of abundance being represented by different cross-hatching (upper panel). **C)** Frequency of occurrence of each category in each temperature treatment. Mean values for each category were obtained from three biological replicates.

Further investigations were performed on seedlings to determine whether the temperature-change effects were influenced by the developmental stage of the host. No *Pst* resistance was observed in seedlings of UC1041 *–Yr36* infected with isolate 08/21, resulting in higher levels of *Pst* uredia than seen on adult plants. As in adult UC1041 plants, there were no significant differences in disease levels between plants which were maintained at either 12°C/18°C or 12°C/25°C pre and post-inoculation (Figure 3A). As in adult plants, enhanced resistance was observed when seedlings were transferred from 12°C/18°C to12°C/25°C (*P < 0.001*, Figure 3A). However, there was no significant difference in uredia levels between plants maintained at 12°C/25°C and plants transferred from 12°C/25°C to 12°C/18°C after *Pst* inoculation, contrary to the reduced resistance observed in adults plants.

*Pst* development and colonisation was observed microscopically in UC1041 seedlings exhibiting enhanced resistance resulting from the transfer to the higher temperature. There were no significant differences in the percentage of germinated urediniospores between the four temperature treatments at both 1 dpi (Additional file 1: Figure S1A) and 3 dpi (Additional file 1: Figure S1C). Similarly, there were no significant differences in the percentage of germinated urediniospores forming SSVs between the four temperature regimes at either 1 dpi or 3 dpi (Additional file 1: Figure S1B,D). At 6 dpi seedlings grown at 12°C/25°C and then transferred to 12°C/18°C post inoculation had significantly smaller internal fungal structures (*P < 0.001*) compared to all other treatments (Additional file 1: Figure S1E). By 8 dpi, hyphal colonisation was less in seedlings grown at 12°C/18°C pre inoculation compared to those grown at 12°C/25°C, regardless of the subsequent temperature change (*P < 0.001*, Figure 3B,C). As expected, seedlings grown at 12°C/18°C and then transferred to the higher temperature regime after *Pst* inoculation showed significantly less hyphal colonisation compared to seedlings maintained at 12°C/18°C (*P < 0.001*). Hyphal growth in seedlings maintained at 12°C/25°C did not differ significantly from seedlings transferred from 12°C/25°C to 12°C/18°C after inoculation (Figure 3B,C).

### Effect of temperature change on *Pst* resistance in wheat cultivars is genotype-dependent

**Figure 4:**
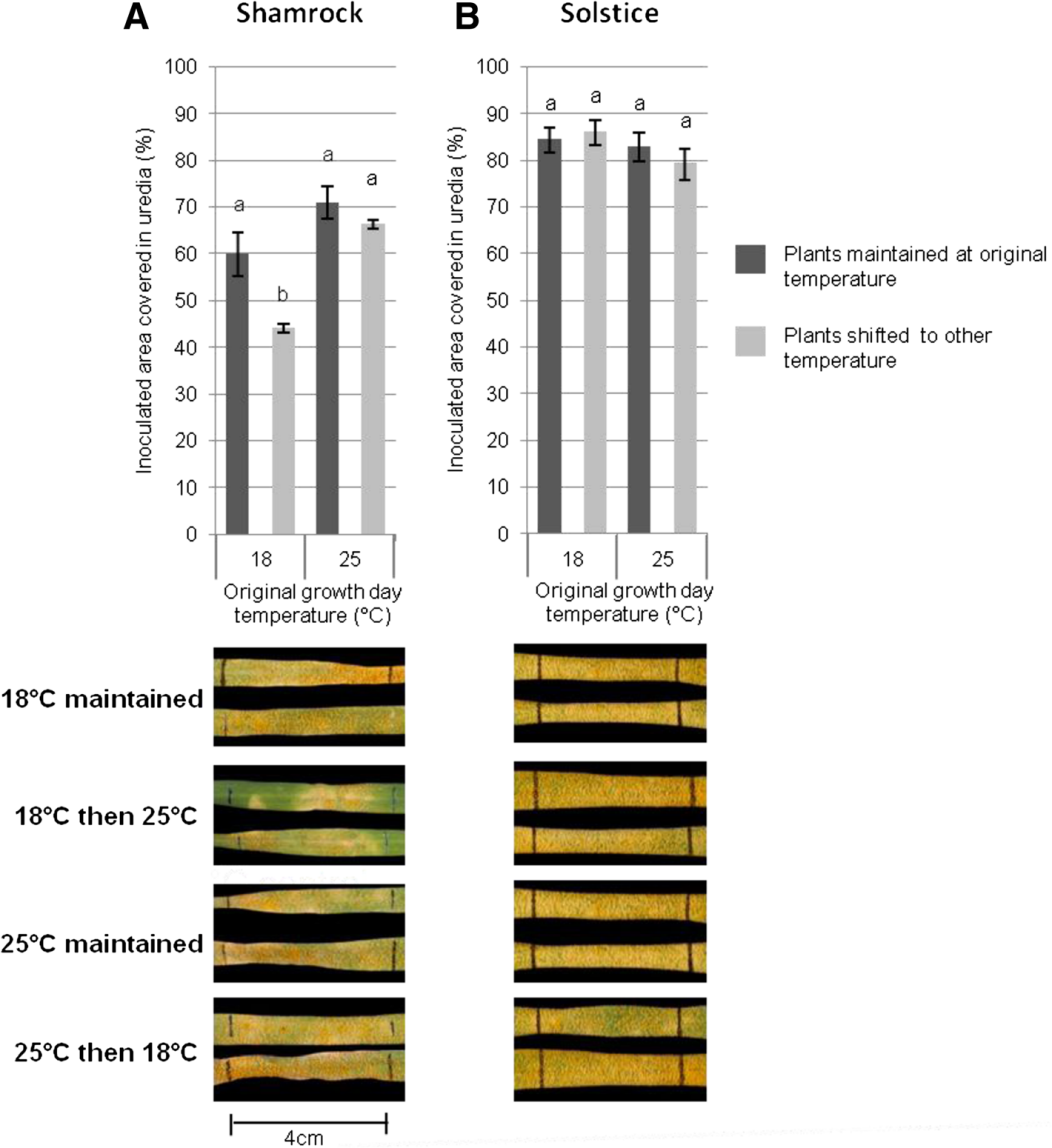
**The effect of changes in temperature on yellow rust resistance in wheat cultivars Shamrock and Solstice.** Percentage (%) leaf area covered in uredia 18 dpi with *Pst* isolate 08/21 when kept at the same temperature regime of 12°C/18°C or 12°C/25°C pre and post inoculation (dark grey) or transferred to the other temperature regime post inoculation (light grey). Wheat cultivars **A)** Shamrock and **B)** Solstice. The *Pst* inoculated area is bounded by black, vertical lines. Mean values (± 1 standard error) were obtained from at least two independent experiments where cultivars Shamrock and Solstice were inoculated at the same time. Different letters indicate statistically significant differences (*P* <0.01).

To determine whether temperature changes induce resistance to *Pst* in other wheat cultivars the same experimental procedures were carried out on seedlings of selected UK elite cultivars. Resistance to *Pst* isolate 08/21 in Shamrock displayed a similar phenotype to UC1041, with seedlings that were transferred from lower to higher temperatures displaying a significant reduction in uredia levels (*P < 0.05*, Figure 4A). However, resistance in Solstice was not affected by transferring between temperature regimes, as disease levels did not differ significantly between all four treatments (Figure 4B). As in UC1041 seedlings, resistance enhancement in Shamrock was not consistent. When seedlings were transferred from 12°C/18°C to 12°C/25°C enhanced resistance was observed in 3 out of 4 experiments.

## Discussion

Our investigations show that *Yr36* confers almost complete resistance to *Pst* isolate 08/21 at 25°C, consistent with previous studies. We also show that *Yr36* is effective at 18°C, refining the lower range of temperature at which resistance is conferred. In addition, we discovered that the UC1041 genetic background responds to changes in temperature independently of *Yr36,* affecting growth of *Pst.* Our results suggest that *Yr36*-mediated resistance may be affected by a previously uncharacterised defence temperature sensitivity which is present in the UC1041background. In the field, *Yr36* conveys a QDR phenotype on adult plants when grown in Mediterranean environments [6]. Based on the results presented here, frequent temperature changes could be influencing the QDR observed for UC1041 + *Yr36* in the field. Furthermore, our results also suggest that *Yr36*-mediated resistance should be effective in the field under relatively cooler temperate conditions.

Several studies characterising temperature-responsive resistance genes have been performed using different pre and post inoculation conditions, but the effect of temperature change has rarely been considered. Yet under natural conditions, temperatures are constantly changing and the effect of this on resistance needs further investigation. It is possible that other temperature-responsive resistance genes may respond to changes in temperature in a similar way to that seen in UC1041 +/- *Yr36*, rather than only requiring exposure to a temperature threshold [10,23,24]. For example, a study by Broers and Wallenburg [12] observed that a decrease in temperature increases *Lr34/Yr18* resistance to leaf rust, although in this study there was no negative control treatment involving plants not exposed to the temperature change. Pretorius *et al*[10] later point out that the Broers and Wallenburg [12] study does not exclude background effects from the cultivars in which the *Lr34/Yr18* gene resided. Our findings highlight the importance of a change in temperature regime in pathology studies and especially when characterising temperature-responsive resistance genes.

Adult plant tests are the most appropriate methods for evaluating resistance, but limit the number experiments that can be performed. Although seedling tests enable more rapid screening and testing of resistance, our results reveal that results are less reliable than with adult plants. Studies with UC1041 seedlings revealed that a temperature increase can enhance resistance to *Pst* at an early stage of plant development. Although the results are less consistent than those observed in adult plants, the results suggest that the effect of temperature changes may not be only due to previously uncharacterised APR resistance in the UC1041 background. Also in seedlings, a decrease in temperature does not reduce resistance to *Pst* as seen in adult plants of UC1041 (40% infection), but this could be due to the higher levels of *Pst* infection seen on UC1041 seedlings (approximately 90% infection). Differences between adult plants and seedlings could be caused by *Pst* inoculum levels, or reflect physiological differences influencing defence, considering some types of resistance cannot be detected until later developmental stages [25]. As in UC1041, the enhanced resistance observed in Shamrock was not seen in all seedling experiments. When the phenotype was not observed, uredia abundance was higher in both cultivars compared to experiments where a temperature effect was observed. This suggests that high levels of *Pst* inoculum and subsequent heavy infection loads may mask the effect of the temperature change.

We show that *Pst* is able to germinate and penetrate the plant successfully, regardless of the temperature-regime to which the plants have been exposed. This would suggest that the temperature-responsive resistance does not involve a mechanism dependent on the initial recognition of the pathogen. The phenotype observed when UC1041 and Shamrock plants were transferred from 12°C/18°C to 12°C/25°C resembles a type of QDR normally associated with decreased infection frequency, increased latency period, and reduced uredium size which generally only show signs of infection later in the growing season [11,26-28]. Some aspects of this phenotype are seen with HTAP yellow rust resistance, which is a QDR generally effective after stem elongation and when day temperatures are 25°C –30°C [7]. However, the enhanced adult plant yellow rust resistance we observed was induced by an increase in temperature rather than prolonged exposure to 25°C, and was also observed in seedlings. Our results therefore suggest that temperature changes, rather than exposure to a threshold temperature, could be influencing some QDR mechanisms.

Plants must continuously adapt to changing environments, and balance resources between growth and defence to achieve maximum productivity [29,30]. Studies in *Arabidopsis thaliana* suggest a general trade-off mechanism whereby hormone-mediated growth may antagonise immune responses [31,32]. Consequently, temperature changes may trigger reorganisation of energy supplies in some wheat cultivars, indirectly resulting in reduced nutrient availability to *Pst*, a biotrophic fungus [33,34]. Another possibility is that temperature changes could lead to the accumulation of pathogen-deterring metabolites [35]. Although QDR mechanisms are largely unknown, *Lr34* and *Yr36* do not fall into the general NBS-LRR class of R genes, so it is reasonable to hypothesise that these are not generally involved in pathogen recognition. Some QDR genes may have other functions that indirectly affect pathogen development when exposed to changes in temperature.

Our results show that a change in temperature up to 8 days before inoculation affected defence against *Pst* in UC1041 adult plants. Thus, pre-exposure to a different temperature regime affects subsequent defence, which suggests an adaptive response. We have no evidence that the temperature change is resulting in a stress response, but the lasting effect of increased or decreased resistance is comparable to priming, whereby previous exposure of plants to stress enables a faster response to subsequent stresses [36]. Ambient temperature changes, similar to the ones used in this study, have been shown to cause adaptive change through epigenetic modification of DNA activity by methylation [37]. It is tempting to speculate that the corresponding temperature changes in wheat could also epigenetically prime plants, affecting later stages of defence, as seen in UC1041.

Seedlings of Shamrock demonstrated a similar resistance phenotype to UC1041 when transferred to a higher temperature post-inoculation. However, resistance in Solstice seedlings was not affected by a temperature change in either direction, indicating that the response is genotype-dependent and that some cultivars may have more stable defence against *Pst* than others under varying temperatures. Park *et al*[38] support these observations, reporting differences between wheat cultivars when challenged with *Pst* at different pre and post inoculation temperature regimes. The authors attributed enhanced resistance at higher temperatures to factors that control adult plant resistance, such as uncharacterised QDR genes being present in the cultivars used in their study. If the basis for our observations is host adaptation to temperature changes, then other pathogen interactions may also be affected. A significant difference in resistance to changing temperatures was observed between wheat cultivars in response to *Blumeria graminis* f. sp *tritici*, the causal agent of wheat powdery mildew, indicating that the phenomenon may not be restricted to yellow rust resistance [39]. Understanding the effects of temperature changes on plant defence will be essential for developing crops that are more resilient to the potential impacts of climate change. Consistent crop performance and reliable disease resistance are important traits in plant breeding. Our results suggest an experimental approach to study the resistance response to changes in temperature. Further investigation will reveal whether this trait interacts with other resistances, and if it can be used to select wheat cultivars with more consistent, temperature-stable resistance to *Pst* and other diseases.

## Conclusions

*Yr36* can prevent *Pst* pustule development at temperatures sustained below 25°C which is contrary to what has previously been shown. In addition, a previously uncharacterised defence temperature sensitivity was discovered in the UC1041 background, revealing that changes in temperature can affect subsequent wheat resistance to yellow rust and may do so in a cultivar dependent manner. Understanding how temperature changes affect resistance could enable the breeding of more stable pathogen resistance in crops.

## Methods

### Plant and pathogen material

Hexaploid wheat near-isogenic lines (NILs) UC1041+/- *Yr36* were obtained from UC Davis, California, USA and cultivars Shamrock and Solstice from the UK Germplasm Resources Unit, held at the Norwich Research Park, UK. All virulence assays were performed with *Pst* isolate 08/21 obtained from the National Institute of Agricultural Botany (NIAB), Cambridge, UK and isolated in the UK in 2008 from the wheat cultivar Solstice. None of the germplasm used in the current study had complete seedling resistance to *Pst* 08/21 as determined in standard seedling tests (data not shown).

### Plant growth conditions

Seeds were sown directly into 1 litre pots and grown to the emergence of the first flag leaf (Zadok’s Growth Stage (GS) 47), or in P15 seed trays for seedling (Zadok’s GS 13-14) assays [40]. Plants were grown in controlled environment rooms (CERs) with an 8 hr/16 hr dark/light cycle, a constant 80% relative humidity and a light intensity of approximately 350 μmol m^-2^ s^-1^. The two diurnal temperature regimes were 12°C/18°C and 12°C/25°C, night/day temperatures (day temperature being the only difference between treatments). To synchronise growth stage, plants were grown one week earlier in the 12°C/18°C regime. For assays on wheat seedlings, plants were sown one to two days earlier in the 12°C/18°C regime, depending on the cultivar.

### Yellow rust inoculations of wheat plants

Inoculations were carried out on flag leaves of adult plants and the newest fully-expanded leaf of seedlings. Plants were inoculated with *Pst* urediniospores exactly 1 hour before the end of the light period. A 4 cm (seedlings) or 5 cm (adult plants) region of the adaxial surface of the leaf was defined and urediniospores were applied with a fine brush as a 1:8 (seedlings) or 1:4 spore/talc mixture (adult plants). The leaf surface was then sprayed with H_2_O containing Tween20 ® (0.01% v/v) to encourage germination. In the UC1041 +/- *Yr36* NIL comparison experiments the same procedure was used to apply urediniospores, but whole leaves were inoculated. In all experiments with the UC1041 *–Yr36* line only, plants were inoculated in a defined area. Plants were then placed in a dew chamber at 12°C, in total darkness for 22 hrs before being returned either to the original temperature regime, or transferred to the new regime. At 18 days post inoculation (dpi) the same 4-5 cm region (or the whole leaf for UC1041 NIL comparisons) was used to determine Percentage infection (Pi), measured as the percentage of leaf tissue (independent of chlorosis or necrosis) covered with sporulating uredia.

### Microscopic analysis of subcellular *Pst* development

Inoculated seedlings of UC1041 were sampled at 1, 3, 6 and 8 dpi. The 4 cm inoculated region of the leaf was harvested and prepared for microscopy using a method adapted from Ayliffe *et al*[41]. For removal of chlorophyll, samples were left to clear overnight in 12 ml of 1 M KOH with 2 μl of Tween20 ® at 37°C. The tissue was rinsed three times in 50 mM Tris at pH 7.5, followed by staining with WGA-FITC at 1 mg/ml in 50 mM Tris for 1 hr. The tissue was mounted on a slide and observed under fluorescent light (465-495 nm > 515-555 nm) using a fluorescence microscope (Nikon 800 Eclipse; Nikon Precision Europe GmbH, Langen, Germany). Samples from early time points were examined for both urediniospore germination and the ability of germinated urediniospores to form sub-stomatal vesicles (SSVs). Later time points were scored by measuring the size of internal fungal structures (μm) and abundance of hyphae in up to 50 fields of view at 10× magnification.

### Data analyses

Data were analysed using the statistical package Genstat for Windows, release 12 (VSN international, Hemel Hempstead, UK). Percentage infection (Pi) scores were transformed using a LOGIT + transformation to obtain near normality [42].

$$\mathrm{LOGI}T^{+}=\log_{n}\left[\frac{\left(\mathrm{Pi}+\left(\min\mathrm{Pi}+0.25\right)\right)}{\left(\left(\max\mathrm{Pi}+0.25\right)-\mathrm{Pi}\right)}\right]$$

Where Log_n_ is natural logarithm and Pi is percentage pustule cover. A general linear regression model was used on the transformed data and outputs from the model provided predicted means where replicate experiments were performed. The effect of temperature regime and experiments (replicates) was accounted for in the model. *Pst* microscopy data were also analysed using a general linear regression model and a LOGIT + transformation of the percentage data.

## Abbreviations

ETI: Effector-triggered immunity; HR: Hypersensitive response; HTAP: High temperature adult plant (resistance); MAMP: Microbe-associated molecular pattern; PAMP: Pathogen-associated molecular pattern; PRR: Pattern recognition receptor; Pst: *Puccinia striiformis* f. sp *tritici*; PTI: PAMP-triggered immunity; QDR: Quantitative disease resistance; SSV: Sub-stomatal vesicle.

## Competing interests

The authors declare that they have no competing interests.

## Authors’ contributions

RB carried out the majority of the experimental work with help from AM for the different cultivar experiments. RB and CR conceived of the study and RB, CR and CU and GM made contributions to conception and experimental design. RB and GM carried out the statistical analysis. HS, SD and LB were involved in revising of the manuscript. All authors read and approved the final manuscript.

## Authors’ information

RB - PhD student, John Innes centre. GM - Post doctoral researcher. AM - Undergraduate student, University of Sheffield. HS - Post doctoral researcher, John Innes Centre. LB - Research group leader, NIAB. CU - Project leader, John Innes Centre. SD - Senior Lecturer in Atmospheric Sciences, UEA. CR - Senior scientist, John Innes Centre.

## Supplementary Material

Additional file 1: Figure S1 *Pst* development in UC1041 (*–Yr36)* seedlings. Seedlings were inoculated with *Pst* isolate 08/21 and kept at the same temperature regime of 12°C/18°C or 12°C/25°C pre and post inoculation (dark grey) or transferred to the other temperature regime post inoculation (light grey). **A)** Percentage of *Pst* urediniospores germinated at 1 dpi. **B)** Percentage of germinated urediniospores forming sub-stomatal vesicles (SSVs) at 1 dpi. **C)** Percentage of *Pst* urediniospores germinated at 3 dpi. **D)** Percentage of germinated urediniospores forming SSVs at 3 dpi. Mean values (± 1 standard error) were obtained from up to 50 fields of view from two independent experiments. Different letters indicate statistically significant differences (*P* <0.01). **E)** Size of *Pst* hyphal colonies (μm) at 6 dpi. Mean values (± 1 standard error) were obtained from three biological replicates. Different letters indicate statistically significant differences (*P* <0.001). Click here for file

## References

[B1] Singh RP Hodson DP Huerta-Espino J Jin Y Bhavani S Njau P Herrera-Foessel S Singh PK Sukhwinder S Govindan V The emergence of Ug99 races of the stem rust fungus is a threat to world wheat production Annu Rev Phytopathol 2011 49 465 481 10.1146/annurev-phyto-072910-095423 21568701

[B2] Markell SG Milus EA Emergence of a novel population of Puccinia striiformis f. sp tritici in eastern United States Phytopathology 2008 98 6 632 639 10.1094/PHYTO-98-6-0632 18944286

[B3] Hovmoller MS Sorensen CK Walter S Justesen AF VanAlfen NK, Bruening G, Leach JE Diversity of *Puccinia striiformis* on cereals and grasses Annual review of phytopathology, Vol 49 2011 197 217 vol. 49 10.1146/annurev-phyto-072910-095230 21599494

[B4] Navabi A Tewari JP Singh RP McCallum B Laroche A Briggs KG Inheritance and QTL analysis of durable resistance to stripe and leaf rusts in an Australian cultivar, Triticum aestivum ‘Cook’ Genome 2005 48 1 97 107 10.1139/g04-100 15729401

[B5] Chen XM Challenges and solutions for stripe rust control in the United States Aust J Agr Res 2007 58 6 648 655 10.1071/AR07045

[B6] Uauy C Brevis JC Chen XM Khan I Jackson L Chicaiza O Distelfeld A Fahima T Dubcovsky J High-temperature adult-plant (HTAP) stripe rust resistance gene *Yr36* from Triticum turgidum ssp dicoccoides is closely linked to the grain protein content locus Gpc-B1 Theor Appl Genet 2005 112 1 97 105 10.1007/s00122-005-0109-x 16208504

[B7] Lin F Chen XM Genetics and molecular mapping of genes for race-specific all-stage resistance and non-race-specific high-temperature adult-plant resistance to stripe rust in spring wheat cultivar Alpowa Theor Appl Genet 2007 114 7 1277 1287 10.1007/s00122-007-0518-0 17318493

[B8] Fu DL Uauy C Distelfeld A Blechl A Epstein L Chen XM Sela H Fahima T Dubcovsky J A kinase-START gene confers temperature-dependent resistance to wheat stripe rust Sci (Washington) 2009 323 5919 1357 1359 10.1126/science.1166289 PMC4737487 19228999

[B9] Plotnikova LY Stubei TY Effectiveness of the wheat *Lr22b, Lr34*, and *Lr37* genes for adult plant resistance to leaf rust in West Siberia and the cytophysiological basis of their action Russ J Genet Appl Res 2013 3 1 47 53 10.1134/S2079059713010115

[B10] Pretorius ZA Kloppers FJ Drijepondt SC Effects of inoculum density and temperature on 3 components of leaf rust resistance controlled by lr34 in wheat Euphytica 1994 74 1–2 91 96

[B11] Rubiales D Niks RE Charaterization of *Lr34*, a major gene conferring nonhypersensitive resistance to wheat leaf rust Plant Dis 1995 79 12 1208 1212 10.1094/PD-79-1208

[B12] Broers LHM Wallenburg SC Influence of postinfection temperature on 3 components of partial resistance in wheat to wheat leaf rust Euphytica 1989 44 3 215 224 10.1007/BF00037528

[B13] Krattinger SG Lagudah ES Spielmeyer W Singh RP Huerta-Espino J McFadden H Bossolini E Selter LL Keller B A putative ABC transporter confers durable resistance to multiple fungal pathogens in wheat Science 2009 323 5919 1360 1363 10.1126/science.1166453 19229000

[B14] Kumar J Jaiswal V Kumar A Kumar N Mir RR Kumar S Dhariwal R Tyagi S Khandelwal M Prabhu KV Introgression of a major gene for high grain protein content in some Indian bread wheat cultivars Field Crop Res 2011 123 3 226 233 10.1016/j.fcr.2011.05.013

[B15] Randhawa HS Asif M Pozniak C Clarke JM Graf RJ Fox SL Humphreys DG Knox RE DePauw RM Singh AK Application of molecular markers to wheat breeding in Canada Plant Breed 2013 132 458 471 n/a-n/a

[B16] Tabbita F Lewis S Vouilloz JP Ortega MA Kade M Abbate PE Barneix AJ Effects of the *Gpc-B1* locus on high grain protein content introgressed into Argentinean wheat germplasm Plant Breed 2013 132 1 48 52 10.1111/pbr.12011

[B17] Zhang Z Feng J Bai Y Lin R Peng Y Xu S Genetic analysis of resistance to stripe rust in wheat cultivar Holdfast J China Agricult Univ 2011 16 5 1 5

[B18] Feng J Zuo LL Zhang ZY Lin RM Cao YY Xu SC Quantitative trait loci for temperature-sensitive resistance to *Puccinia striiformis f. sp tritici* in wheat cultivar Flinor Euphytica 2011 178 3 321 329 10.1007/s10681-010-0291-z

[B19] Wan A Niu Y Wu L Temperature-sensitive resistance to yellow rust in 22 wheat cultivars Sci Agric Sin 2000 33 101 103

[B20] Zipfel C Early molecular events in PAMP-triggered immunity Curr Opin Plant Biol 2009 12 4 414 420 10.1016/j.pbi.2009.06.003 19608450

[B21] Schwessinger B Ronald PC Merchant SS Plant innate immunity: perception of conserved microbial signatures Annual review of plant biology, Vol 63 2012 451 482 vol. 63 10.1146/annurev-arplant-042811-105518 22404464

[B22] Jones JDG Dangl JL The plant immune system Nature 2006 444 323 329 10.1038/nature05286 17108957

[B23] Ramage RA Sutherland MW High and low pre-inoculation temperatures decrease the effectiveness of the *Lr20* and *Sr15* rust resistance genes in wheat Plant Pathol 1995 44 5 772 778 10.1111/j.1365-3059.1995.tb02734.x

[B24] Dyck PL Johnson R Temperature sensitivity of genes for resistance in wheat to *Puccinia recondita* Can J Plant Pathol 1983 5 4 229 234 10.1080/07060668309501601

[B25] Basnet BR Singh RP Herrera-Foessel SA Ibrahim AMH Huerta-Espino J Calvo-Salazar V Rudd JC Genetic analysis of adult plant resistance to yellow rust and leaf rust in common spring wheat Quaiu 3 Plant Dis 2013 97 6 728 736 10.1094/PDIS-02-12-0141-RE 30722591

[B26] Shah SJA Imtiaz M Hussain S Phenotypic and molecular characterization of wheat for slow rusting resistance against *Puccinia striiformis* Westend. f.sp *tritici* J Phytopathol 2010 158 6 393 402

[B27] Herrera-Foessel SA Singh RP Huerta-Espino J Rosewarne GM Periyannan SK Viccars L Calvo-Salazar V Lan CX Lagudah ES *Lr68*: a new gene conferring slow rusting resistance to leaf rust in wheat Theor Appl Genet 2012 124 8 1475 1486 10.1007/s00122-012-1802-1 22297565

[B28] William HM Singh RP Huerta-Espino J Palacios G Suenaga K Characterization of genetic loci conferring adult plant resistance to leaf rust and stripe rust in spring wheat Genome 2006 49 8 977 990 10.1139/G06-052 17036073

[B29] Mosher S Moeder W Nishimura N Jikumaru Y Joo S-H Urquhart W Klessig DF Kim S-K Nambara E Yoshioka K The lesion-mimic mutant cpr22 shows alterations in abscisic acid signaling and abscisic acid insensitivity in a salicylic acid-dependent manner Plant Physiol 2010 152 4 1901 1913 10.1104/pp.109.152603 20164209 PMC2850030

[B30] Koga H Dohi K Mori M Abscisic acid and low temperatures suppress the whole plant-specific resistance reaction of rice plants to the infection of *Magnaporthe grisea* Physiol Mol Plant Pathol 2004 65 1 3 9 10.1016/j.pmpp.2004.11.002

[B31] Albrecht C Boutrot F Segonzac C Schwessinger B Gimenez-Ibanez S Chinchilla D Rathjen JP de Vries SC Zipfel C Brassinosteroids inhibit pathogen-associated molecular pattern-triggered immune signaling independent of the receptor kinase BAK1 Proc Natl Acad Sci U S A 2012 109 1 303 308 10.1073/pnas.1109921108 22087006 PMC3252947

[B32] Anderson JP Badruzsaufari E Schenk PM Manners JM Desmond OJ Ehlert C Maclean DJ Ebert PR Kazan K Antagonistic interaction between abscisic acid and jasmonate-ethylene signaling pathways modulates defense gene expression and disease resistance in *Arabidopsis* Plant Cell 2004 16 12 3460 3479 10.1105/tpc.104.025833 15548743 PMC535886

[B33] Viola R Davies HV Effect of temperature on pathways of carbohydrate-metabolism in tubers of potato (*Solanum-tuberosum L*) Plant Sci 1994 103 2 135 143 10.1016/0168-9452(94)90201-1

[B34] Grof CPL Campbell JA Kravchuk O Lambrides CJ Albertson PL Temperature effect on carbon partitioning in two commercial cultivars of sugarcane Funct Plant Biol 2010 37 4 334 341 10.1071/FP09216

[B35] Hu B Simon J Rennenberg H Drought and air warming affect the species-specific levels of stress-related foliar metabolites of three oak species on acidic and calcareous soil Tree Physiol 2013 33 5 489 504 10.1093/treephys/tpt025 23619385

[B36] Conrath U Beckers GJM Flors V Garcia-Agustin P Jakab G Mauch F Newman M-A Pieterse CMJ Poinssot B Pozo MJ Priming: getting ready for battle Mol Plant Microbe Interact 2006 19 10 1062 1071 10.1094/MPMI-19-1062 17022170

[B37] Kumar SV Wigge PA H2A.Z-containing nucleosomes mediate the thermosensory response in *Arabidopsis* Cell 2010 140 1 136 147 10.1016/j.cell.2009.11.006 20079334

[B38] Park RF Ash GJ Rees RG Effects of temperature on the response of some Australian wheat cultivars to *Puccinia-striiformis* f-sp *tritici* Mycol Res 1992 96 166 170 10.1016/S0953-7562(09)80961-1

[B39] Ge YF Johnson JW Roberts JJ Rajaram S Temperature and resistance gene interactions in the expression of resistance to *Blumeria Graminis f. sp. Tritici* Euphytica 1998 99 2 103 109 10.1023/A:1018392725474

[B40] Zadoks JC Chang TT Konzak CF Decimal code for growth stages of cereals Weed Res 1974 14 6 415 421 10.1111/j.1365-3180.1974.tb01084.x

[B41] Ayliffe M Jin Y Kang Z Persson M Steffenson B Wang S Leung H Determining the basis of nonhost resistance in rice to cereal rusts Euphytica 2011 179 1 33 40 10.1007/s10681-010-0280-2

[B42] Powell NM Lewis CM Berry ST MacCormack R Boyd LA Stripe rust resistance genes in the UK winter wheat cultivar Claire Theor Appl Genet 2013 126 1599 1612 10.1007/s00122-013-2077-x 23536048

