# Supplementary figures and images for: A change in temperature modulates defence to yellow (stripe) rust in wheat line UC1041 independently of resistance gene Yr36
Source: BMC Plant Biol. 2014 Jan 8;14:10. doi: 10.1186/1471-2229-14-10 (PMC3898064; doi:10.1186/1471-2229-14-10)

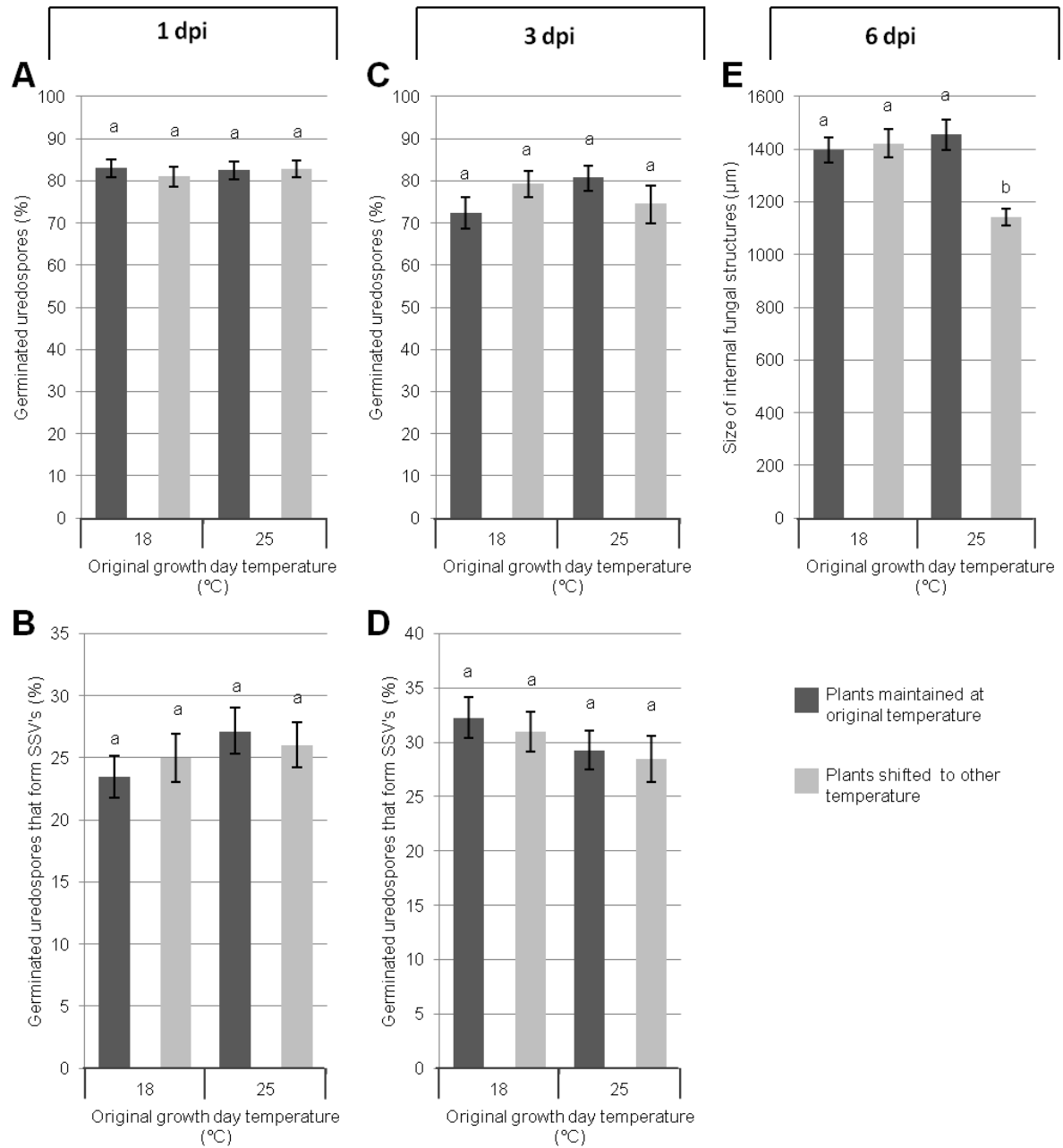

Supplement: Additional file 1: Figure S1 — Pst development in UC1041 (–Yr36) seedlings. Seedlings were inoculated with Pst isolate 08/21 and kept at the same temperature regime of 12°C/18°C or 12°C/25°C pre and post inoculation (dark grey) or transferred to the other temperature regime post inoculation (light grey). A) Percentage of Pst urediniospores germinated at 1 dpi. B) Percentage of germinated urediniospores forming sub-stomatal vesicles (SSVs) at 1 dpi. C) Percentage of Pst urediniospores germinated at 3 dpi. D) Percentage of germinated urediniospores forming SSVs at 3 dpi. Mean values (± 1 standard error) were obtained from up to 50 fields of view from two independent experiments. Different letters indicate statistically significant differences (P <0.01). E) Size of Pst hyphal colonies (μm) at 6 dpi. Mean values (± 1 standard error) were obtained from three biological replicates. Different letters indicate statistically significant differences (P <0.001). [file 1471-2229-14-10-S1.pdf]
